# Demonstration and biological significance of a gastrin‐P21‐activated kinase 1 feedback loop in colorectal cancer cells

**DOI:** 10.14814/phy2.12048

**Published:** 2014-06-24

**Authors:** Nhi Huynh, Kevin H. Liu, Mildred Yim, Arthur Shulkes, Graham S. Baldwin, Hong He

**Affiliations:** 1 Department of Surgery, University of Melbourne, Austin Health, Melbourne, Victoria, Australia

**Keywords:** Colon cancer, Gamide, glycine extended, p21‐activated kinase 1

## Abstract

Gastrins, including amidated gastrin_17_ and glycine‐extended gastrin_17_, are important growth factors in colorectal cancer (CRC). The p21‐activated kinase 1 (PAK1) plays key roles in cellular processes including proliferation, survival, and motility, and in cell transformation and tumor progression. PAK1 expression increases with the progression of CRC, and knockdown of PAK1 blocks CRC cell growth and metastasis both in vitro and in vivo. The aim of this study was to determine the interaction between PAK1 and gastrins in CRC cells. PAK1 expression and activation were assayed by Western blots, and concentrations of gastrin mRNA and peptides by real‐time PCR and radioimmunoassay, respectively. Proliferation of CRC cells was measured by ^3^H‐thymidine incorporation, and vascular endothelial growth factor **(**VEGF) secretion was measured by ELISA. Gastrins activated PAK1 via PI3K‐dependent pathways. Activated PAK1 in turn mediated gastrin‐stimulated activation of *β*‐catenin and VEGF secretion in CRC cells, as knockdown of PAK1 blocked stimulation of these cellular processes by gastrins. Downregulation of gastrin reduced the expression and activity of PAK1, but in contrast there was a compensatory increase in gastrins either when PAK1 was downregulated, or after treatment with a PAK inhibitor. Our results indicate that PAK1 is required for the stimulation of CRC cells by gastrins, and suggest the existence of an inhibitory feedback loop by which PAK1 downregulates gastrin production in CRC cells.

## Abstract (precis)

The results in this study indicate that PAK1 is required for the stimulation of CRC cells by gastrins, and suggest the existence of an inhibitory feedback loop by which PAK1 downregulates gastrin production in CRC cells.

## Introduction

Colorectal cancer (CRC) arises and progresses as a result of cumulative genetic and epigenetic changes in tumor cells. Mutations in the Ras and Wnt/*β*‐catenin signaling pathway*s* occur in 50% and 90% of CRCs (Kolligs et al. 2002; Yuen et al. 2002), respectively. Constitutive activation of Wnt/*β*‐catenin signaling initiates the growth of benign adenomas, while mutations in KRas, BRaf, and related pathways stimulate adenoma growth and contribute to invasive and other malignant behaviors. Aberrant activation of *β*‐catenin promotes cell proliferation and survival, and initiates colorectal tumorigenesis (He et al. 1998; Polakis 2007). Dissociation of *β*‐catenin from E‐cadherin at the cell membrane triggers the loss of adherens junctions, which causes the epithelial‐to‐mesenchymal transition, a change in cellular phenotype associated with the progression of cancers of epithelial origin (Thiery 2002). Accumulation of nuclear *β*‐catenin, and the consequent formation of a constitutively active complex with the transcription factor T‐cell factor 4 (TCF4; Korinek et al. 1997; Morin et al. 1997), promotes cell growth and drives tumor progression through upregulation of target genes such as c‐myc (He et al. 1998; van de Wetering et al. 2002). The nuclear translocation and activation of *β*‐catenin is suppressed by inhibition of p21‐activated kinase 1 (PAK1) in CRC cells (He et al. 2012), and PAK1 stimulates CRC cell growth in vitro and in vivo by activation of *β*‐catenin.

p21‐activated kinase 1 is a serine/threonine kinase that functions as a downstream node for normal and oncogenic signaling pathways (Kumar et al. 2006). PAK1 was originally identified as a protein that interacts with Rac and Cdc42, which are members of the Rho family of G proteins. PAK1 regulates cytoskeletal remodeling and cell motility, and mediates growth factor‐stimulated cell migration, proliferation, and cancer cell metastasis. PAK1 is essential for malignant transformation induced by Ras, Rac, and Cdc42 (Tang et al. 2000; He et al. 2001; Kumar et al. 2006), and enhances transformation through the ERK/MAPK signaling pathway (He et al. 2000; Bokoch 2003). Activation of PAK1 is required for growth of CRC cells (Huynh et al. 2010; Zhu et al. 2011). Furthermore, PAK1 signaling is required for vascular endothelial growth factor (VEGF) expression and function, and consequently for angiogenesis (Bagheri‐Yarmand et al. 2000; Huynh et al. 2010), which in turn promotes tumor growth and metastasis. Although PAK1 has been implicated in many growth factor‐stimulated signaling pathways, the role of PAK1 in the regulation of CRC progression by gastrins has not been investigated.

The gastrins are a family of gastrointestinal peptide hormones, which include both amidated (Gamide) and glycine‐extended (Ggly) forms. In gastrointestinal cancer, gastrin gene expression is upregulated by aberrant activation of the Wnt/*β*‐catenin and Ras pathways (Koh et al. 2000; Lei et al. 2004; Chakladar et al. 2005). Colorectal tumors produce gastrins which in turn stimulate the proliferation of tumor cells (Seva et al. 1994; Iwase et al. 1997; Ferrand and Wang 2006). Both Gamide and Ggly have been implicated in stimulation of cell proliferation (Seva et al. 1994; Taniguchi et al. 1996; Koh and Chen 2000; Singh et al. 2000; Smith and Watson 2000; Todisco et al. 2001), inhibition of apoptosis, and acceleration of neoplastic transformation (Taniguchi et al. 1996; Koh and Chen 2000; Smith and Watson 2000; Todisco et al. 2001). Gamide, acting via the cholecystokinin‐2 (CCK‐2) receptor, activates Jun N‐terminal kinase (JNK) and p38 MAPK through a PKC‐dependent pathway (Dehez et al. 2001, 2002), and induces the phosphorylation of the adaptor protein Shc, which associates with the complex Grb2/Sos, leading to the activation of the Ras/Raf/MEK/ERK cascade (Seva et al. 1996; Daulhac et al. 1997). Gamide also stimulates a PI3K/AKT‐dependent pathway (Daulhac et al. 1999; Ferrand et al. 2004). Ggly activates JNK and PI3K/AKT‐dependent pathways to regulate cell proliferation and apoptosis (Hollande et al. 2001; Beales and Ogunwobi 2006). The small GTP binding proteins Rho, Rac, and Cdc42 are required for the regulation of cell proliferation, migration, and apoptosis by both Gamide and Ggly (Stepan et al. 2004; He et al. 2005).

Our recent study has demonstrated that PAK1 mediated gastrin‐stimulated proliferation in normal murine colorectal mucosa via multiple signaling pathways in vivo (Huynh et al. 2013). The facts that gastrins are growth factors involved in CRC development, and that inhibition of PAK1 blocks CRC cell growth and metastasis in vitro and in vivo (He et al. 2012), suggest that PAK1 may play a role in the regulation of CRC progression by gastrins. To investigate the role of PAK1 in the stimulation of CRC development by gastrins, the effect of PAK1 knockdown on gastrin‐stimulated activation of *β*‐catenin, and secretion of VEGF, was measured in CRC cells. The effects of PAK1 inhibition on gastrin production by CRC cells, and of gastrin knockdown on PAK1 expression and activity, have also been investigated in vitro. Here, we report the existence of an inhibitory feedback loop by which PAK1 downregulates gastrin production in CRC cells.

## Materials and Methods

### Reagents, cell culture, and transfection

Amidated (Gamide) and glycine‐extended (Ggly) gastrin_17_ were synthesized by Auspep (Melbourne, Vic., Australia). SureSilencing shRNA plasmids for human PAK1 were purchased from SABioscience (Frederick, MD). Antibodies against PAK1, phospho‐AKT, AKT, E‐cadherin, *β*‐catenin, c‐myc, and GAPDH were purchased from Cell Signalling Technology (Genesearch, Melbourne, Vic., Australia). The antiphospho‐PAK1 antibody was purchased from Santa Cruz Biotechnology (Santa Cruz, CA). Human VEGF ELISA kits were purchased from R&D systems (Minneapolis, MN). The PI3K inhibitor LY294002 and the PAK inhibitor PF3758309 were bought from Sigma (St. Louis, MO) and from Active Biochemical (Maplewood, NJ), respectively.

The human CRC cell lines DLD1 and HCT116, which were obtained from ATCC and tested negative for mycoplasma, were cultured in RPMI or DMEM medium containing 5% FBS. To obtain PAK1 knockdown cells, DLD1 and HCT116 cells were transfected with plasmid DNAs encoding shRNA sequences to silence the PAK1 gene specifically, or with a scrambled sequence as a negative control (NC) as described previously (He et al. 2012). Constitutively active (CA, PAK1^T423E^) and wild‐type (WT) PAK1 constructs (generously provided by Dr. Gary Bokoch, Scripps Research Institute, La Jolla, CA) were subcloned into the pCDNA3.1 vector (Invitrogen, Melbourne, Vic., Australia) as described previously (Liu et al. 2013). CA‐ and WT‐PAK1 plasmid DNAs were transiently transfected into DLD1 cells using Lipofectin Reagent (Invitrogen) according to the manufacturer's instructions.

### Cell proliferation assay

Cell proliferation was assayed by ^3^H‐thymidine incorporation. Cells were seeded in a 96‐well plate at a density of 5 × 10^3^ cells/well in growth medium containing 5% FBS and 10 *μ*Ci/mL [methyl‐^3^H]‐thymidine and cultured at 37°C for 24 h. The cells were then harvested using a cell harvester (Nunc, Roskilde, Denmark). The amount of ^3^H‐thymidine incorporated through DNA synthesis was detected with a *β*‐counter (Packard, Meriden, CT).

### Luciferase assays

*β*‐catenin/TCF4 transcriptional activity was determined by luciferase assay. Cells, 1 × 10^6^, were transfected with either 5 *μ*g of TOP‐Flash plasmid (given by Dr. Randall T. Moon, Howard Hughes Medical Institute, Chevy Chase, MD) and 0.5 *μ*g of pGL4.1 *β*‐gal reporter (Promega, Madison, WI), as internal control, using a Neon Transfection System (Invitrogen). Transfected cells were seeded in a 24‐well plate at 1 × 10^5^ cells/well in DMEM containing 1% FBS in the presence or absence of either Gamide (10 nmol/L) or Ggly (10 nmol/L). Luciferase activity was measured using the dual‐luciferase reporter assay system (Promega) following the manufacturer's protocol, and activity was normalized to *β*‐gal reporter activity.

### VEGF assay

Cells were cultured in six‐well plates in RPMI without serum for 48 h in the absence or presence of Gamide (10 nmol/L) or Ggly (10 nmol/L). The media were then collected, cleared by centrifugation and the VEGF concentrations determined in duplicate at a 1:4 dilution using a VEGF ELISA kit (R&D systems) following the manufacturer's instructions and corrected for total cell numbers.

### Immunoblotting

Cells were lysed in lysis buffer (50 mmol/L HEPES, pH 7.5, 150 mmol/L NaCl, 5 mmol/L MgCl_2_, 1% NP40, 1 mmol/L DTT, 5 *μ*g/mL aprotinin, 5 *μ*g/mL leupeptin, and 1 mmol/L PMSF). The cell lysates were centrifuged at 13,000 *g* for 10 min at 4°C, and the protein concentration from the resultant supernatants was quantified using Bradford reagent (Sigma). In some cases, the supernatants were immunoprecipitated with anti‐*β*‐catenin antibody, followed by immunoblotting. For immunoblotting samples from either cell lysates or immunoprecipitates were resolved by SDS‐PAGE, and proteins were detected with the antibodies indicated in the text. The bound antibodies were visualized using ECL reagents (GE Healthcare, Amersham, Buckinghamshire, UK), and the density of each band was analyzed using Multigauge computer software (Berthold, Bundoora, Vic., Australia).

### Quantitative real‐time PCR

To measure gastrin mRNA, cells were seeded at 4 × 10^5^/well in six‐well plates, and total RNA was extracted with TRIzol (Invitrogen) and converted to cDNA using Superscript^™^ III first strand synthesis system (Invitrogen). The resulting cDNA transcripts were used for real‐time PCR amplification with the ABI 7700 Sequence Detector (Applied Biosystems, Melbourne, Vic., Australia) and Taqman chemistry according to the manufacturer's instructions. Primer pairs for gastrin were 5′‐CCG CAG TGC TGA AGA TGA G‐3′ and 5′‐GGA GGT GGC TAG GCT CTG AA‐3′ (Kovac et al. 2010). Results were normalized to 18S RNA expression.

### Gastrin radioimmunoassay

Five million cells were seeded and serum starved for 24 h before protein extraction. Gastrins from the cell extracts were determined by radioimmunoassay (RIA) as described previously (Ciccotosto et al. 1995). Region‐specific gastrin antisera were used to measure Gamide (antiserum 1296), Ggly (antiserum 7270), and progastrin (antiserum 1137).

### Statistical analysis

All values are expressed as means ± standard error. Results were analyzed by one‐way analysis of variance. If there was a statistically significant difference in the data set, individual values were compared by Bonferroni's *t* test with the unstimulated control, or with the values obtained in the presence of gastrins. Differences between two means with *P *<**0.05 were considered significant.

## Results

### Gastrins activate PAK1 via a PI3K‐dependent pathway in CRC cells

**Figure 1.**
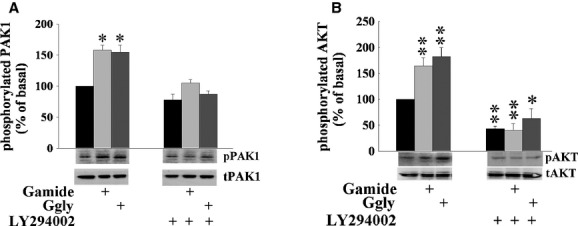
Gastrins stimulate p21‐activated kinase 1 (PAK1) activity via PI3K‐dependent pathways. Wild‐type DLD1 cells were preincubated with or without the PI3K inhibitor, LY294002 (10 μmol/L) for 1 h, followed by incubation with or without Gamide (10 nmol/L) or Ggly (10 nmol/L) for 10 min. (A) PAK1 activity was determined as the ratio of active phosphorylated PAK1 (pPAK1) to total PAK1 (tPAK1) measured by Western blotting as described in Materials and Methods. The value for control cells treated with neither LY294002 nor gastrins was taken as 100%. (B) AKT activity was determined as the ratio of active phosphorylated AKT (pAKT) to total AKT (tAKT) measured by Western blotting as described in Materials and Methods. The value for control cells treated with neither LY294002 nor gastrins was taken as 100%. The results represent data summarized from three independent experiments. **P < 0.01; *P < 0.05 compared to the values from untreated control cells.

To determine whether gastrins stimulate PAK1 activity in CRC cells, DLD1 cells were stimulated with Gamide or Ggly for 10 min in the presence or absence of LY294002, a PI3K inhibitor, and the activity of PAK1 was determined by measuring the ratio of the phosphorylated and active form of PAK1 to total PAK1 by Western blot. Both Gamide and Ggly significantly increased the phosphorylation of PAK1 to 150% of control (Fig. 1A), and stimulation by either gastrin was blocked by the PI3K inhibitor, LY294002. Neither Gamide nor Ggly affected total PAK1 expression during the 10‐min incubation (Fig. 1A). Both Gamide and Ggly also increased the phosphorylation of AKT, the downstream effector of PI3K, to more than 150% of the control value (Fig. 1B), and stimulation by either gastrin was blocked by LY294002, which also inhibited the basal phosphorylation of AKT. These results indicate that both Gamide and Ggly activate PAK1 through PI3K‐dependent pathways.

### Downregulation of PAK1 is associated with reduced proliferation in gastrin‐antisense‐transfected CRC cells

**Figure 2.**
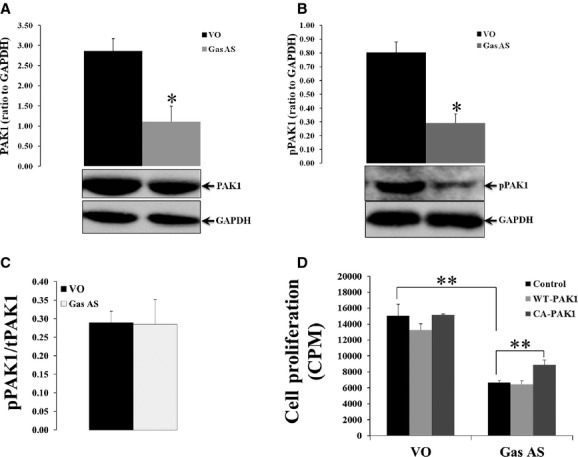
Downregulation of gastrin decreases proliferation and p21‐activated kinase 1 (PAK1) expression and activity in colorectal cancer (CRC) cells. Lysates from vector‐only (VO) and gastrin‐antisense (Gas AS)‐transfected DLD1 cells were blotted with antibody to total PAK1 (A), phosphorylated PAK1 (B), or GAPDH. The ratios of phosphorylated PAK1 (pPAK1) to total PAK1 (tPAK1) from VO and Gas AS cells were compared (C). Both VO and Gas AS cells were transiently transfected with vector alone (control), or with vector encoding wild‐type (WT) PAK1 or constitutively active (CA) PAK1 and proliferation was assayed by ^3^H‐incorporation (D). The results represent data summarized from three independent experiments. **P < 0.01; *P < 0.05 compared with VO transfected (A, B) or as indicated (D).

To determine the effect of inhibition of gastrins on PAK1 in CRC cells, the expression and activity of PAK1 were measured in DLD1 cells in which endogenous gastrin production had been reduced by stable transfection with an antisense gastrin plasmid (Gas AS; Hollande et al. 2003). Downregulation of gastrin in DLD1 cells decreased PAK1 protein expression (Fig. 2A) and activation (Fig. 2B) by over 50% compared to vector‐only transfected (VO) cells. The fact that the ratio of pPAK1 to PAK1 remained unchanged (Fig. 2C) suggests that the overall reduction of PAK1 activation in Gas AS cells was due to the decreased protein expression of total PAK1 in these cells. These data indicate that gastrins stimulate the activation of PAK1 via promoting its protein expression in CRC cells as depletion of gastrin resulted in a similar reduction of PAK1 protein expression and activation.

Furthermore, the proliferation of Gas AS cells was decreased compared to the VO cells (Fig. 2D). Transient transfection of WT or CA PAK1 did not affect the proliferation of VO cells. However, overexpression of CA‐PAK1 significantly increased the proliferation of Gas AS cells (Fig. 2D). The fact that an increase in PAK1 activity could partially rescue cells after gastrin knockdown is consistent with the suggestion that gastrins promote CRC cell proliferation at least partially through activation of PAK1.

### PAK1 knockdown blocks gastrin‐stimulated activation of β‐catenin and VEGF production in CRC cells

**Figure 3.**
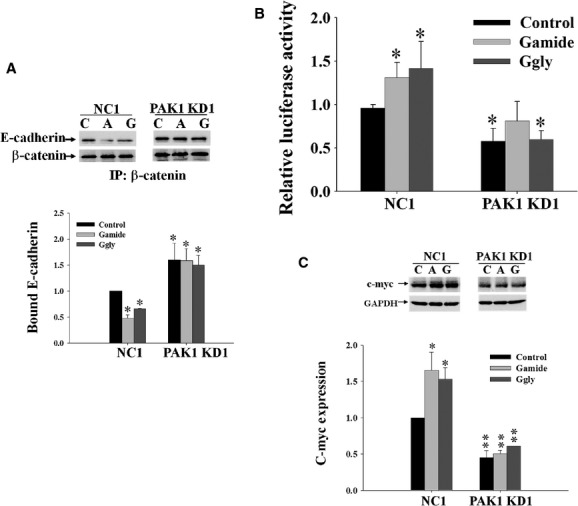
p21‐activated kinase 1 (PAK1) knockdown blocks gastrin‐stimulated activation of *β*‐catenin. DLD1 cells were transfected with plasmid DNAs containing either shRNA insert sequences to knock down PAK1 (PAK1 KD) or scrambled sequences to serve as a negative control (NC). (A) Gastrin‐induced dissociation of *β*‐catenin from E‐cadherin. Both NC and PAK1 KD cells were incubated without (control, C) or with Gamide (A, 10 nmol/L) or Ggly (G, 10 nmol/L) for 24 h. The cells were lysed and the cell lysates were immunoprecipitated (IP) with anti‐*β*‐catenin antibodies followed by immunoblotting with either anti‐Ε‐cadherin or anti‐*β*‐catenin antibodies. Protein expression in the untreated NC cells was taken as 1. (B) Gastrin‐stimulated *β*‐catenin/T‐cell factor 4 (TCF4) transcriptional activity. Both NC1 and PAK1 KD1 cells were stimulated with either Gamide (10 nmol/L) or Ggly (10 nmol/L) for 24 h, and then subjected to luciferase assays as described in Materials and Methods to determine the transcriptional activity of *β*‐catenin/TCF4. The relative luciferase activity of NC cells was taken as 1. (C) Gastrin‐induced c‐myc expression. The cell lysates from A were directly immunoblotted to determine the expression of c‐myc. Protein expression was normalized to GAPDH, and the value for untreated NC cells was taken as 1. Figures B, and the bottom panels in A and C, represent the data summarized from three independent experiments. *P < 0.05; **P < 0.01 compared with the values for untreated NC cells.

To determine the role of PAK1 in the stimulation of CRC cell by gastrins, DLD1 cells were transfected with plasmid DNAs containing either shRNA insert sequences to knock down (KD) PAK1 or scrambled sequences to serve as a NC. Among four insert sequences provided by the manufacturer (SABioscience), PAK1 KD clones were successfully selected from cells transfected with insert sequences 2 and 3. The selected PAK1 KD clones had PAK1 protein expression <10% of the value in the NC cells transfected with scrambled sequences (Huynh et al. 2010). To determine the effect of PAK1 knockdown on the gastrin‐stimulated activation of *β*‐catenin, the association of *β*‐catenin with E‐cadherin, the transcriptional activity of *β*‐catenin/TCF4, and the expression of c‐myc were measured by immunoprecipitation, luciferase assay, and Western blot, respectively, as described in Materials and Methods. After 24‐h incubation, both Gamide and Ggly reduced the amount of E‐cadherin bound to *β*‐catenin in NC cells (Fig. 3A). Significantly more E‐cadherin was bound to *β*‐catenin in PAK1 KD cells than in NC cells. The reduction of E‐cadherin bound to *β*‐catenin induced by gastrin was blocked in PAK1 KD cells (Fig. 3A). Similarly, Gamide and Ggly also stimulated the transcriptional activity of *β*‐catenin/TCF4 in NC cells (Fig. 3B). PAK1 knockdown not only reduced the transcriptional activity of *β*‐catenin/TCF4 but also blocked the stimulation by gastrins of the transcriptional activity of *β*‐catenin/TCF4 (Fig. 3B). Furthermore, both Gamide and Ggly stimulated the expression of c‐myc in NC cells, and PAK1 knockdown reduced the expression of c‐myc and blocked the stimulation of c‐myc expression by gastrins (Fig. 3C). These results indicate that PAK1 is required for both intrinsic and gastrin‐stimulated activation of *β*‐catenin.

**Figure 4.**
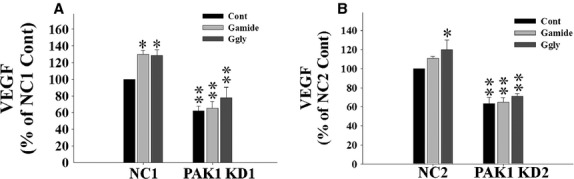
p21‐activated kinase 1 (PAK1) knockdown blocks gastrin‐stimulated vascular endothelial growth factor (VEGF) secretion. NC1 and PAK1 KD1 (A), or NC2 and PAK1 KD2 (B), DLD1 cells were incubated with or without Gamide (10 nmol/L) or Ggly (10 nmol/L) for 48 h. VEGF production in the conditioned media was measured by ELISA. The results represent data summarized from three independent experiments where VEGF production by untreated negative control (NC) cells was taken as 100%. The raw value of VEGF for NC1 cells in the absence of gastrins was 208 pg/10^5^ cells. *P < 0.05; **P < 0.01 compared with untreated NC cells.

In CRC, VEGF expression correlates with cancer stage and prognosis, and VEGF stimulates angiogenesis which further promotes tumor survival and metastasis (Ishigami et al. 1998; Kumar et al. 1998; Tokunaga et al. 1998; Ellis et al. 2000). To determine the effects of PAK1 on the regulation of CRC cell survival by gastrins, VEGF production by both PAK1 KD and NC cells in the absence or presence of gastrins was measured by ELISA. After 48 h of culture, VEGF production from PAK1 KD cells was significantly lower than from NC cells (Fig. 4). Both Gamide and Ggly stimulated the production of VEGF by NC cells, but gastrin‐stimulated VEGF production was blocked in PAK1 KD cells (Fig. 4). These results indicate that PAK1 is essential for both intrinsic and gastrin‐stimulated VEGF production.

### Inhibition of PAK1 increases gastrin production in CRC cells

**Figure 5.**
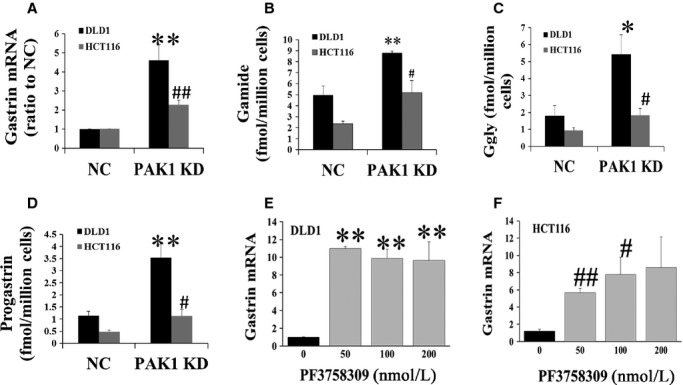
Reduced p21‐activated kinase 1 (PAK1) expression increases gastrin production. Gastrin mRNA (A)**,** or Gamide (B), Ggly (C) or progastrin (D) peptides, in extracts of negative control (NC) or PAK1 KD DLD1 or HCT116 cells were measured by real‐time PCR (A) or radioimmunoassay (B–D) as described in Materials and Methods. The concentration of gastrin mRNA in extracts from DLD1 (E) or HCT116 (F) cells after treatment with the indicated concentrations of the PAK inhibitor PF3758309 was also determined by real‐time PCR. The results represent data summarized from six independent experiments. *P < 0.05; **P < 0.01, compared with values from NC DLD1 cells (A–D) or untreated DLD1 cells (E); ^#^*P* < 0.05; ^##^*P* < 0.01, compared with values from NC HCT116 cells (A–D) or untreated HCT116 cells (F).

**Figure 6.**
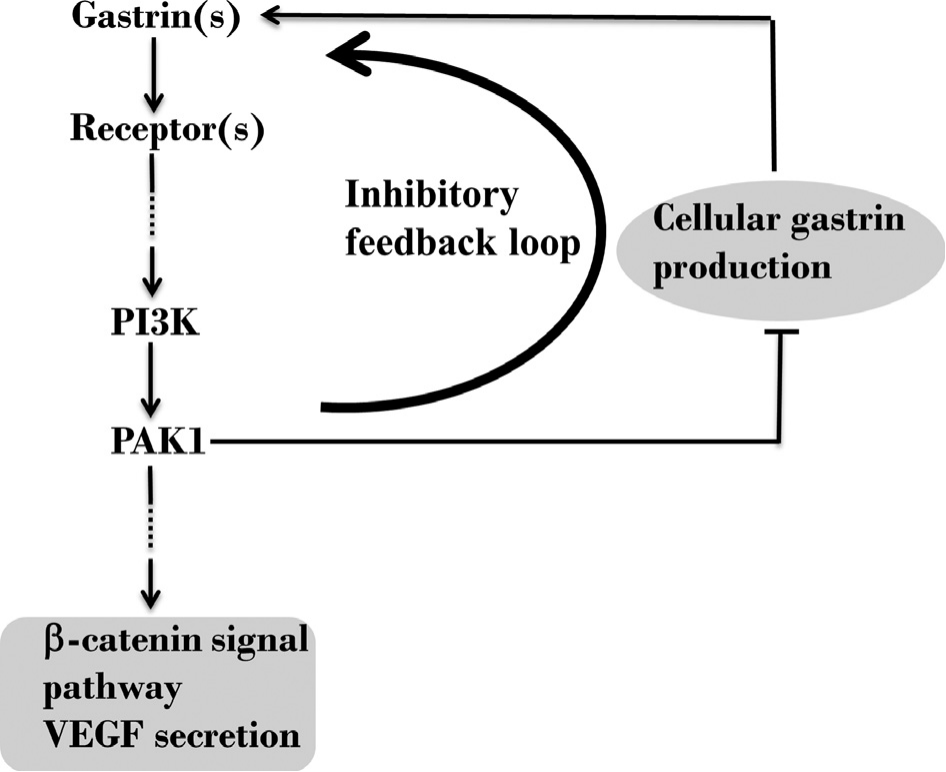
p21‐activated kinase 1 (PAK1) mediates stimulation of colorectal cancer (CRC) cells by gastrins and negatively regulates gastrin production. Gastrins stimulate PAK1 expression and activity via activation of PI3K, and PAK1 mediates, at least in part, the effects of gastrins on CRC cells. Down‐regulation of PAK1 increases gastrin production via reduction of an inhibitory feedback loop. Hence, CRC cells may partly compensate for the functions lost as a result of PAK1 knockdown by upregulation of gastrins.

To investigate the effect of PAK1 knockdown on gastrin production by CRC cells, gastrin mRNA and peptides in extracts of both PAK1 KD and NC DLD1 cells were measured by real‐time PCR and by RIA, respectively, as described in Materials and Methods. Gastrin mRNA was significantly increased in PAK1 KD cell extracts to nearly 2.5 times the value in NC cell extracts (Fig. 5A). Similarly, Gamide in PAK1 KD cell extracts was increased to almost twice the value in NC cell extracts (Fig. 5B), and Ggly and progastrin in PAK1 KD cell extracts were both increased to nearly three times the value in NC cell extracts (Fig. 5C and D). Similar results were also obtained in HCT116 cells (Fig. 5A–D). These data showed that downregulation of PAK1 in CRC cells resulted in a significant increase in cellular gastrin production. Furthermore, inhibition of PAK1 by PF3758309, a nonselective PAK inhibitor with Ki values of 14 nmol/L and 19 nmol/L for the isolated kinase domains of PAK1 and PAK4, respectively (Murray et al. 2010), significantly increased gastrin mRNA in both DLD1 (Fig. 5E) and HCT116 cells (Fig. 5F). The doses used here were based on the IC_50_ values (≈100 nmol/L) for inhibition of proliferation of these CRC cell lines by PF‐3758309 (data not shown). These results suggest the existence of an inhibitory feedback loop whereby an increase in PAK1 expression/activation reduces cellular gastrin production (Fig. 6).

## Discussion

p21‐activated kinase 1 enhances proliferation, migration/invasion, and survival of CRC cells by activation of ERK and AKT, as knockdown of PAK1 by small interfering RNA (siRNA) dramatically reduced cell proliferation, migration/invasion, and survival, as well as the activities of ERK and AKT (Huynh et al. 2010). PAK1 is also required for activation of the *β*‐catenin signaling pathway in CRC cells, as PAK1 knockdown reduced *β*‐catenin expression and inhibited the transcriptional activity of *β*‐catenin/TCF4 (He et al. 2012). Together these findings indicate that PAK1 is involved in the activation of multiple signaling pathways that are important for CRC growth and metastasis.

Our recent in vivo study has showed that proliferation in the normal colonic and rectal mucosa was reduced in PAK1 knockout (KO) mice compared to wild‐type controls (Huynh et al. 2013). Moreover, the decreased proliferation in the colorectal mucosa was associated with reduced activation of ERK, AKT, and *β*‐catenin signaling pathways. On the other hand, PAK1 expression and activity were inhibited in the colorectal mucosa of gastrin KO mice, and the inhibition was associated with reduced proliferation and decreased activation of ERK, AKT, and *β*‐catenin signaling pathways. Together these findings indicate that PAK1 mediated gastrin‐stimulated proliferation of normal colorectal mucosa in vivo by activation of multiple signaling pathways. Consistent with the in vivo findings, gastrin‐stimulated proliferation and migration was also blocked by PAK1 knockdown (KD) in the human CRC cell line DLD1 (Huynh et al. 2013). Here, we have further demonstrated that PAK1 was activated in human CRC cell lines in vitro by both Gamide and Ggly through PI3K‐dependent pathways, and that the activation of PAK1 contributed to gastrin‐stimulated activation of *β*‐catenin signaling and VEGF production in CRC cells, as PAK1 knockdown completely blocked these stimulatory effects of gastrins on CRC cells.

Both Gamide (Ferrand et al. 2004) and Ggly (Hollande et al. 2001; He et al. 2008) stimulate migration of gastric epithelial cells via PI3K‐dependent pathways. Ggly also stimulates CRC cell proliferation via both MAPK/ERK‐ and PI3K‐dependent pathways (Ferrand et al. 2006), and inhibits CRC cell apoptosis by activation of PI3K, Janus kinase 2 (JAK2), and JNK pathways (Beales and Ogunwobi 2006). PI3K plays an important role in CRC progression (Khaleghpour et al. 2004), and inhibition of PI3K by siRNA treatment suppresses CRC cell growth in vitro and as metastatic tumors in mice (Rychahou et al. 2006). PI3K can phosphorylate and activate PAK1 (Papakonstanti and Stournaras 2002), which functions as a key node in various signaling pathways leading to cell survival, migration, and growth. With respect to the effects of gastrins, our observations that Gamide and Ggly activate PAK1 through PI3K‐dependent pathways identify PAK1 as a crucial downstream effector of PI3K, responsible for mediation of PI3K‐activated signaling. We have also shown here that PAK1 mediates gastrin‐stimulated activation of *β*‐catenin (Fig. 3) and VEGF production in CRC cells (Fig. 4), as knockdown of PAK1 blocked the stimulatory effects of gastrins on both activation of *β*‐catenin and VEGF production. These in vitro data demonstrate that PAK1, activated by gastrins via a PI3K‐dependent pathway, mediates the stimulatory effects of gastrins on *β*‐catenin signaling and VEGF production, which in turn play a crucial role in the proliferation, migration/invasion, and survival of CRC cells.

Knockdown of PAK1 by siRNA also resulted in increased cellular gastrin production at both the mRNA and peptide levels in two human CRC cell lines (Fig. 5), an observation consistent with our previous findings in PAK1 KO mice (Huynh et al. 2013). This conclusion was further strengthened by the observation that treatment with the nonselective PAK inhibitor PF‐3758309, increased gastrin mRNA in two CRC cell lines. These results suggest the existence of a novel inhibitory feedback loop whereby an increase in PAK1 activity reduces gastrin expression (Fig. 6). Even though gastrins stimulate cell proliferation and migration (Huynh et al. 2013), and *β*‐catenin signaling and VEGF production (Fig. 3), in CRC cells, the upregulation of gastrins via removal of the inhibitory feedback loop demonstrated in this study does not compensate for the reduction in these functions as a result of PAK1 knockdown.

On the other hand, both protein expression and activation of PAK1 were decreased in gastrin‐antisense‐transfected cells (Gas AS), with an associated decrease in proliferation (Fig. 2). Overexpression of CA PAK1, but not WT PAK1, partially reversed the reduction of proliferation in Gas AS cells. Together these findings further strengthen the conclusion that gastrins promote the expression and activation of PAK1, which plays a key role in mediating gastrin stimulation of CRC cells. In relation to our findings here, it will be interesting to measure the concentration and activity of PAK1, as well as gastrins and progastrin in tumors from CRC patients since we and others have reported that circulating gastrins and progastrin were increased in CRC patients (Do et al. 2012; Paterson et al. 2014).

This study has demonstrated that gastrins activate PAK1 via a PI3K‐dependent pathway, and that activation of PAK1 contributes to gastrin‐stimulated activation of *β*‐catenin and VEGF production in CRC cells. We have also identified the existence of an inhibitory feedback loop whereby PAK1 activation suppresses gastrin production in CRC cells. We conclude that activated PAK1 plays key roles in mediating production of gastrins by, and their stimulation of, CRC cells.
